# esloco: simulation-based estimation of local coverage in long-read DNA sequencing

**DOI:** 10.1093/bioinformatics/btag009

**Published:** 2026-01-09

**Authors:** Adrian Weich, Christopher Lischer, Julio Vera

**Affiliations:** Department of Dermatology, Friedrich-Alexander-Universität (FAU) Erlangen-Nürnberg and Uniklinikum Erlangen, 91054 Erlangen, Germany; Comprehensive Cancer Center Erlangen-European Metropolitan Area of Nuremberg (CCC ER-EMN), 91054 Erlangen, Germany; Deutsches Zentrum Immuntherapie (DZI), Erlangen, Germany; Department of Internal Medicine 5, Hematology and Oncology, Friedrich-Alexander-Universität (FAU) Erlangen-Nürnberg and Uniklinikum Erlangen, 91054 Erlangen, Germany; Department of Dermatology, Friedrich-Alexander-Universität (FAU) Erlangen-Nürnberg and Uniklinikum Erlangen, 91054 Erlangen, Germany; Comprehensive Cancer Center Erlangen-European Metropolitan Area of Nuremberg (CCC ER-EMN), 91054 Erlangen, Germany; Deutsches Zentrum Immuntherapie (DZI), Erlangen, Germany

## Abstract

**Summary:**

Long-read DNA sequencing is increasingly applied for whole-genome studies, yet experimental planning often lacks reliable estimates of target region coverage, leading to costly and time-consuming pilot studies and replicates. We present *esloco, a* Monte Carlo-based simulation framework for estimating local coverage in long-read sequencing experiments, including scenarios with unknown target regions (e.g. viral integration, CRISPR-Cas9) or PCR-free designs (e.g. base modifications). By modeling coverage as a function of sequencing depth and read length distribution, *esloco* enables informed predictions of local sequencing outcomes. Benchmarking across a 45-gene panel demonstrated close agreement with empirical data, underscoring the framework’s reliability.

**Availability and implementation:**

*esloco* is a Python package available on PyPI (https://pypi.org/project/esloco/), GitHub (https://github.com/aweich/esloco), and Zenodo (https://doi.org/10.5281/zenodo.17776161).

## 1 Introduction

Long-read DNA sequencing (LRS) offers several advantages over traditional short-read sequencing, including the ability to span structural variations, resolve repetitive regions, and generate contiguous genome assemblies (Logsdon *et al.* 2020). While LRS lowers global depth requirements, it introduces variability at the local coverage level. This poses challenges for detecting rare insertions or ensuring adequate coverage of regions of interest (ROIs), such as genes or functional elements that cannot be PCR-amplified due to biological (e.g. repetitive DNA) or experimental (e.g. base modifications) constraints.

A major limitation in experimental planning is the lack of reliable *a priori* estimates of ROI coverage in a sequencing run (Sims *et al.* 2014). In practice, researchers often rely on costly, time-consuming pilot studies, where sequencing is performed first and local coverage assessed afterwards. This trial-and-error approach is inefficient and does not guarantee that sufficient sequencing depth will be achieved across all target regions. Existing web-based tools (Read Coverage Calc | NEB n.d. or Sequencing Support—Coverage Calculator n.d.) assist with whole-genome coverage estimations or sequencing logistics but generally lack the flexibility needed for complex and application-specific scenarios.

To address this, we developed *esloco*, a simulation framework that predicts expected sequencing depth at user-defined or randomized target regions. The simulation models the inherent variability in local coverage based on predefined whole-genome coverage levels and read length distributions. Our approach further allows to assess how different sequencing parameters, e.g. mean read length, whole-genome coverage, and clonality, affect regional sequencing depth.

By providing computationally efficient and data-driven predictions, *esloco* reduces experimental uncertainty, improves cost efficiency, and enhances the design and interpretation of LRS studies.

## 2 Implementation

### 2.1 Simulation structure

The simulation follows a Monte Carlo (Kroese and Rubinstein 2012, Kroese *et al.* 2014) scheme, in which we assume that the whole-genome coverage and the underlying read length distribution of sequencing data primarily determine the coverage of a target region. Our simulation tool *esloco* is implemented as a Python package to ensure flexibility and ease of use. It supports extensive customization through a single configuration file (Table 1, available as supplementary data at *Bioinformatics* online), while remaining straightforward to execute from the command line. All iterative steps are fully parallelized and run exclusively on the CPU, making *esloco* efficient and accessible even on standard computing systems without specialized hardware.

Generally, *esloco* supports two operational modes, i.e. insertion mode (I) and region-of-interest mode (ROI). “I” mode simulates dynamic elements such as transposons or viral insertions, which may vary between individual genomes. “ROI” mode, on the other hand, focuses on predefined, static regions shared across all simulated genomes.

To simulate heterogeneous cell populations, *esloco* introduces artificial “barcodes,” mimicking the concept of molecular barcoding in sequencing experiments. In practice, this means that each simulated genome can be assigned a unique identifier, allowing for both homogeneous (single-barcode, *n* = 1) or heterogeneous (multi-barcode, *n* > 1) simulations.

**Figure 1:**
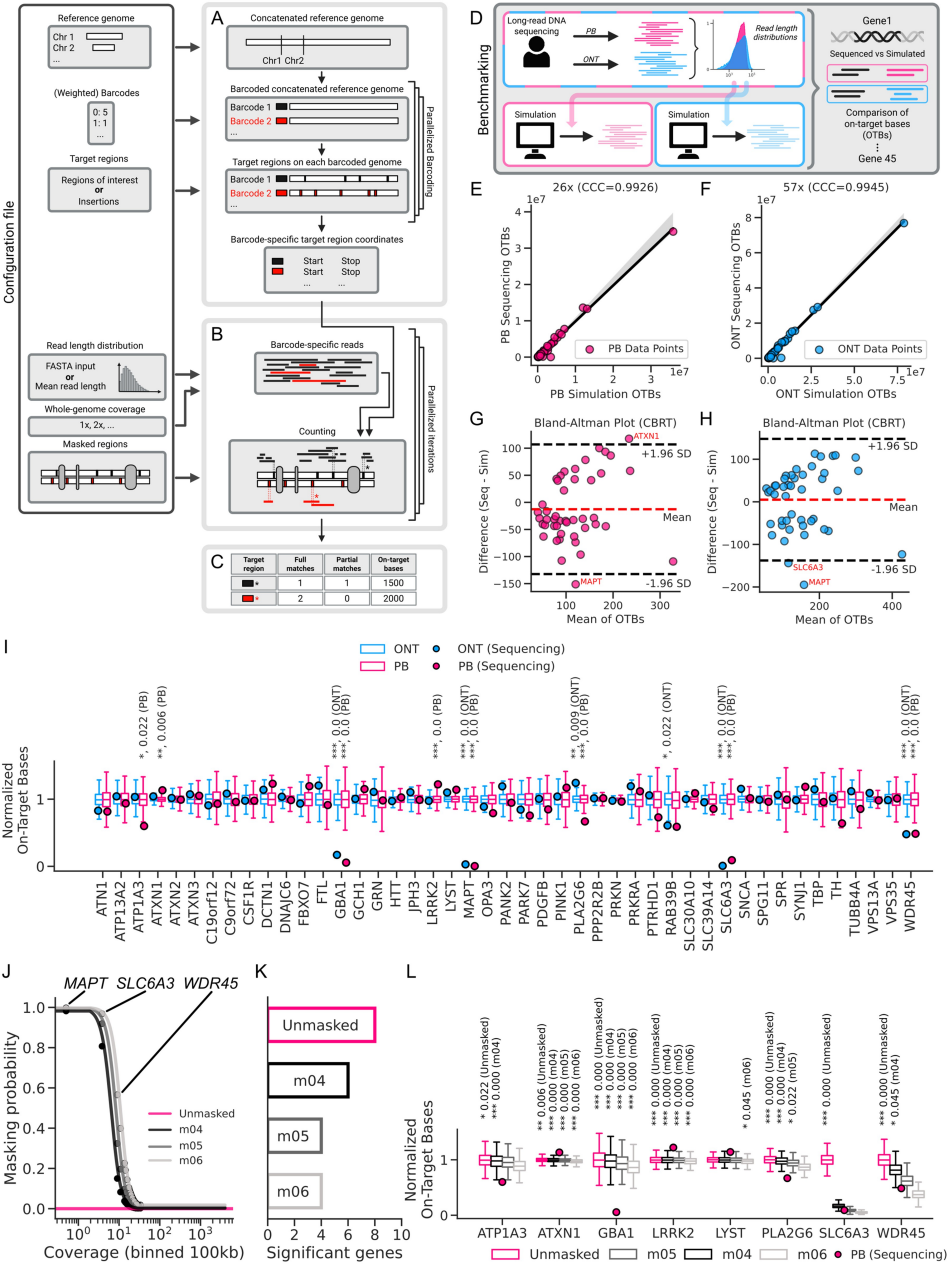
Overview of *esloco* (A–C), benchmarking analysis (D–I), and weighted masking (J–L). (A) Barcode-specific target coordinates are derived either from user-defined regions of interest or from randomly placed insertions in a concatenated reference genome. (B) Reads are drawn from user-defined or empirical read length distributions and placed randomly until the target whole-genome coverage is reached. (C) Local coverage is calculated from overlaps with target regions, excluding reads in masked regions. (D) Benchmarking against empirical local coverage data for a panel of 45 genes. Empirical data came from PacBio (PB) and Oxford Nanopore Technologies (ONT) long-read sequencing of the same biological donor. Sequencing-derived coverage was compared with simulation-derived coverage. Concordance correlation coefficients (CCC) peaked at 26× for PB (E) and 57× for ONT (F). (G, H) Cube-root-transformed (CBRT) coverage values showed strong agreement and no systematic bias for both platforms. Genes outside ±1.96 SD are labeled. (I) Simulated (boxplots) and empirical (points) coverage in OTBs across panel genes and platforms, normalized by gene-length and empirical whole-genome coverage. Eight PB and six ONT genes showed significant deviations between sequenced and simulated values (two-sided empirical *P*-values, Benjamini–Hochberg FDR). (J) Mapping probability of 100 kb-spanning regions and corresponding mean coverage for different *m* values. Mean coverage per bin was used to generate weights for *esloco*. (K) Number of significantly different genes after simulations with masked regions. (L) Predicted and observed coverage for genes previously identified as significantly different. With weighted masking (*m *= 0.5 or *m *= 0.6), only four genes remained significantly different (two-sided empirical *P*-values, Benjamini–Hochberg FDR).

The workflow implemented in the simulation involves generating the genome and targets, simulating sequencing reads, and calculating local coverages over target regions. Each of these steps is outlined below (Fig. 1A–C).

### 2.2 Genome and target region generation

#### 2.2.1 Creation of a barcoded insertion genome (I mode)

The input reference genome is converted into a concatenated sequence while preserving chromosomal coordinates. To distinguish individual genomes, barcode prefixes, i.e. substrings, are added, generating distinct sets of chromosomal coordinates. Insertions are then introduced into each barcoded genome. If an insertion occurs upstream of an existing one, all global insertion coordinates are adjusted accordingly.

The number of insertions (*n_I_*) can either be fixed or drawn from a Poisson distribution around the provided number.


(1) $$n_{I}∼\mathrm{Poisson}(n_{I})$$

Similarly, an insertion’s placement can be entirely random or constrained to a predefined set of potential locations (“semi-random”). If semi-random placement is chosen, the probability of an insertion occurring within a specific region is determined by its relative size:


(2) $$P(\mathrm{Insertion})=\frac{\mathrm{Regionlength}}{\sum \left(\mathrm{Allregionlengths}\right)}$$

Alternatively, probabilities can be assigned to each potential location, enabling more complex insertion distributions.

#### 2.2.2 Creation of a barcoded ROI genome (ROI mode)

Similarly, a concatenated reference genome is used to create barcode-specific chromosomal coordinates of target regions. In contrast to the insertion mode, user-defined ROIs are constant, meaning that all barcodes share the same target regions. If contributions of individual barcodes are unimportant, such pseudo-homogeneous simulations can often be improved performance-wise by using only one barcode.

### 2.3 Simulating sequencing reads

#### 2.3.1 Read length distribution

We assume that reads obtained via LRS follow a long-tailed log-normal distribution (De Coster *et al.* 2018, Logsdon *et al.* 2020). Thus, the tool generates a log-normal distribution around a user-defined mean read length.


(3) $$L_{i}∼\mathrm{LogNormal}(\mu ,\sigma^{2})$$

The standard deviation of the underlying normal distribution (σ) can be chosen by the user to model the highly skewed nature of read lengths. Alternatively, the user can provide a set of reads in FASTA/FASTQ format as input, which is then used as read length distribution.

### 2.4 Read allocation

For each iteration, a read length *L_i_* is sampled from the user-defined or empirical read length distribution *D_L_*.


(4) $$L_{i}∼D_{L}$$

The read is then assigned to a barcode, either uniformly at random or based on user-specified weighting, to reflect varying contributions of individual genomes in mixed samples. Next, the read is placed at random along the span of the corresponding barcode-specific reference genome *G*. Before being accepted, the read’s coordinates are checked against any user-defined masked regions. If the read overlaps such a region, it is discarded. Whether a discarded read contributes to the sequencing yield is controlled by the parameter *consuming*.

This sampling and placement process is repeated until the combined number of read lengths satisfies the desired whole-genome coverage *C_genome_*:


(5) $$\sum_{i=1}^{N}L_{i}\geq C_{\mathrm{Genome}}\cdot G$$

### 2.5 Calculating local coverages

Each barcode-specific read *i* (R_i_) is then checked for full overlaps, partial overlaps, as well as on-target bases (OTBs) with the barcode-specific target regions *r* (T_r_).

Partial matches are defined as reads overlapping with the target regions ($\max(T_{r,\mathrm{start}},R_{i,\mathrm{start}})<\min(T_{r,\mathrm{end}},R_{i,\mathrm{end}})$), but not fully containing them ($R_{i,\mathrm{start}}\leq T_{r,\mathrm{start}}\;\wedge \;T_{r,\mathrm{end}}\leq R_{i,\mathrm{end}}$). OTBs are defined as the number of bases of a read overlapping with the target region ($\min(T_{r,\mathrm{start}},R_{\mathrm{end}})-\max(T_{r,\mathrm{end}},R_{\mathrm{start}})$). Importantly, a match is registered only if the read meets the user-defined criteria for scaling and minimum overlapping bases between the target region and the read. This results in the following definition for the OTBs for one target region *r* and iteration:


(6) $${\mathrm{OTB}}_{r}=\sum_{i=1}^{N}\left|R_{i}\cap T_{r}\right|$$

with ∣R_i_ ∩ T_r_∣ being the number of OTBs from read *i* (*R_i_*) that overlap target region *r* (*T_r_*). Over *M* Monte Carlo iterations, the OTB estimate for one target region *r* can then be defined as:


(7) $$\hat{{OTB}_{r}}=\frac{1}{M}\sum_{j=1}^{M}{\mathrm{OTB}}_{r}^{\left(j\right)}$$

Here, ${\mathrm{OTB}}_{r}^{j}$ is the OTBs estimate from the *j*th simulation iteration. To maintain full control over the analysis, the simulation output includes all results and metrics for each simulation iteration, as well as summary statistics (mean, standard deviation, standard error, and confidence intervals). For convenience, *esloco* includes a downstream analysis code (*plot_esloco*) that automatically visualizes the output within a brief analysis report (exemplary report in Supplementary Data, available as supplementary data at *Bioinformatics* online).

### 2.6 Benchmarking simulated coverage estimations against empirical data

In our validation study, we assessed whether the simulated local coverage for a panel of genes correlates with the empirically observed during LRS. Whole-genome LRS data generated using PacBio (PB) (SRR8955270) (Wang *et al.* 2019) were mapped to the human reference genome (hg38) using minimap2 (v2.26) (Li 2018) using the “map-pb” specification. Only high-confidence primary alignments (mapping quality > 30), excluding secondary, supplementary, and low-quality reads (SAM flag filter: -F 2304) were retained. This resulted in 6 763 580 reads with a mean read length of 11 835 bases. Global depth of coverage of ∼25.8× was calculated using:


(8) $$\mathrm{Coverage}=\frac{L_{\mathrm{mean}}\cdot N_{\mathrm{totalreads}}}{{\mathrm{Genome}}_{\mathrm{haploid}}}$$

To assess generalizability, we also used Oxford Nanopore Technologies (ONT) LRS data from the same biological donor (HG006, Chinese Father, GM24694, Release 2025.1). Raw reads were basecalled using dorado (v1.1.1) with model dna_r10.4.1_e8.2_400bps_hac@v5.2.0 and aligned to the reference genome (hg38) using minimap2 (v2.26) (Li 2018) with the “map-ont” specification. After filtering, 10 939 410 reads with a mean read length of 15 296 bases and a global depth of coverage ∼54× were observed.

We selected genes listed in Parkinson’s Green List (v1.127) as an exemplary gene panel (Martin *et al.* 2019) (Table 2, available as supplementary data at *Bioinformatics* online). To calculate the true positive coverage for all listed genes, we counted the on-target bases (OTBs) in the sequencing data using samtools (v1.19) (Danecek *et al.* 2021) and bedtools (Quinlan and Hall 2010) intersect (-wo).

We then ran tailored simulations for both PB and ONT using their respective empirical read length distributions and whole-genome coverages: 20–30× for PB and 53–58× for ONT (configuration files in Supplementary Data, available as supplementary data at *Bioinformatics* online).

For both platforms, we performed 1000 iterations for each coverage and calculated OTBs in the simulation data for each panel gene, using the mean across all iterations. We computed the significant differences between simulation and sequencing for each panel gene’s number of OTBs using two-sided empirical *P*-values, followed by Benjamini–Hochbergs’s false-discovery-rate (FDR) correction for multiple testing (Benjamini and Hochberg 1995). Additionally, concordance correlation coefficients (Lin 1989) were calculated and Bland-Altman (Altman and Bland 1983) plots were generated.

To address mapping biases, we repeated a subset of the benchmarking simulations using a weighted mask (configuration file in Supplementary Data, available as supplementary data at *Bioinformatics* online). This mask was constructed based on differences in local coverage observed in the empirical PB LRS data. First, we calculated read coverage at each position using bedtools genomecov with the “bga” option. We then created 32 191 bins of 100 kb each using bedtools makewindows and calculated the mean coverage per bin using bedtools map. The binned coverage values were winsorized at the 90th and 10th quantiles to reduce the influence of extreme values, log-transformed (log10), and percentile-scaled. Finally, we mapped the resulting values to blocking probabilities using a logistic function:


(9) $$P(\mathrm{Blocking})=\frac{1}{e^{10(x-m)}}$$

where *m* varied between 0.4, 0.5, and 0.6 to control the midpoint of the logistic curve. This process translated the empirical LRS coverage patterns into a genome-spanning BED file with representative weights. Finally, we repeated the simulation for PB at 26× with 500 iterations for each *m* setting.

## 3 Results

### 3.1 Simulation architecture

First, the chromosomal coordinates of the input reference genome are transformed into a consecutive concatenated reference. For each barcode representing an individual genome in a mixture, we assign a set of target regions and record their barcode-specific locations. The simulation allows users to define target regions in two ways: by providing a BED-like file specifying ROIs (e.g. genes) or by (semi-) randomly placing insertions as target regions (e.g. gene therapy vectors, viruses) (Fig. 1A).

Second, for each combination of whole-genome coverage values and mean read lengths, the simulation samples read lengths from a specified read length distribution. This distribution can either be generated using a log-normal model centered around the mean read length, or supplied as an empirical distribution from a FASTA/FASTQ file. Reads are then randomly placed within the barcode-specific genome range until reaching the desired whole-genome coverage, while reads landing in blocked regions are discarded (Fig. 1B).

Third, for each target region, overlapping reads and their on-target bases (OTBs) are counted to determine the final coverage (Fig. 1C). An overview of *esloco’s* runtime performance across different parameters [such as number of (weighted) barcodes, coverage, iterations, masked regions, and target length] is provided in Fig. 1, available as supplementary data at *Bioinformatics* online.

### 3.2 Reliable estimates for local coverages across a panel of genes

We evaluated the simulation tool using genes associated with Parkinson’s disease. For this, we collected high-quality, high-coverage LRS data generated using PB and ONT and compared the sequencing results to a specifically tailored simulation conducted using *esloco* (Fig. 1D). For PB, the simulation completed 1000 iterations across selected coverage conditions (20–30×) in ∼91 h, using 50 CPU cores with a peak memory usage of 79.54 MB per iteration. For ONT, the simulation completed 1000 iterations across selected coverage conditions (53–58×) in ∼95 h, using 50 CPU cores with a peak memory usage of 65.89 MB per iteration.

To assess accuracy, we compared OTBs from sequencing and simulation data across global simulated coverage ranging from 20× to 30× for PB and 53× to 58× for ONT. Concordance correlation coefficients (CCC) suggested 26× for PB (Fig. 1E) and 57× for ONT (Fig. 1F) as the best-fit coverages (CCC_PB_ = 0.993, CCC_ONT_ = 0.9945), with only marginal variation observed at neighboring levels (Fig. 2, available as supplementary data at *Bioinformatics* online). This indicates high correlation and precision between simulated and sequenced coverage levels across panel genes for both platforms. Notably, the best-fit coverage for PB was observed to be very close to the empirical coverage (25,8× versus 26×), while for ONT the best-fit coverage was slightly higher in the simulation (54× versus 57×).

Bland-Altman plots, calculated using cube-root-transformed OTB values at 26× for PB and 57× for ONT, both showed that 95.56% (43/45) of the data points fell within ±1.96 standard deviations, demonstrating the strong agreement between simulation and sequencing across platforms (Fig. 1G and H).

At the simulated sequencing depth achieving the highest CCC for PB (26×), 8 of 45 (17.8%) panel genes showed significantly different simulated OTB counts compared to sequencing (*ATP1A3, ATXN1, GBA1, LRRK2, MAPT, PLA2G6*, *SLC6A3, WDR45*) (Fig. 1I). At the simulated sequencing depth achieving the highest CCC for ONT (57×), 6 of 45 (13.3%) panel genes were significantly different in their simulated OTB counts compared to sequencing (*GBA1*, *MAPT*, *PLA2G6, RAB39B, SLC6A3*, *WDR45*) (Fig. 1I).

Notably, *GBA1, MAPT, and SLC6A3* were consistently overestimated by the simulations for both platforms. Further investigation revealed that this discrepancy was likely due to the mapping quality filter: almost all sequencing reads covering these three genes were excluded during pre-processing (Fig. 3, available as supplementary data at *Bioinformatics* online). As a result, there was an artificially introduced mismatch between simulated and sequenced coverage for these three genes, highlighting how downstream processing steps, such as read alignment and quality filtering, can affect the reliability of simulation predictions.

To address this, we repeated the simulation at 26× coverage using a weighted mask based on the empirical PB data (Fig. 1J). This reduced the number of significantly different genes to only four for *m *= 0.5 and *m *= 0.6 (Fig. 1K). Importantly, *MAPT*, *SLCA3*, and *WDR45* were no longer significantly different (Fig. 1L), indicating that their discrepancies were indeed driven by mapping-bias. Additionally, no new significantly different genes emerged following masking, demonstrating the robustness of the approach (Fig. 4, available as supplementary data at *Bioinformatics* online).

However, the discrepancy for *GBA1* remained unsolved. *GBA1* (10 247 bp) lies within the 100 kb bin *chr1: 155 200 000–155 300 000*, for which the logistic function assigned a blocking probability of only 0.048 (for *m *= 0.5). Further examination revealed that coverage across this bin was highly heterogeneous, ranging from >30× to 0× (Fig. 5, available as supplementary data at *Bioinformatics* online). Because the bin-wide mean coverage does not reflect the true coverage of *GBA1*, the masking could not produce an accurate blocking probability for this gene.

## 4 Conclusion

The planning of LRS experiments often lacks reliable *a priori* coverage estimates for target regions, and existing tools are too simplistic for application-specific scenarios. We designed *esloco* as a flexible framework that supports user-defined read length distributions, (weighted) barcoding, and (weighted) region masking, enabling users to model the unique characteristics of their sequencing setup. At the same time, *esloco* remains fully functional with minimal input, making it well-suited for exploratory analyses and experimental planning.

While *esloco* assumes uniform read placement, this simplification represents a potential limitation, as biological and technical biases, such as GC content and chromatin state are known to influence sequencing coverage (Ross *et al.* 2013, Delahaye and Nicolas 2021, Chen *et al.* 2025). However, by supporting weighted region masking, *esloco* allows users to incorporate empirical correction factors, offering a route to bias-aware customization.

In summary, *esloco* provides an efficient and adaptable tool for predicting local coverage in LRS, supporting both *a priori* experimental design and *post-hoc* interpretation. Additionally, it offers a platform for exploring questions that would otherwise require complex or impractical experimental setups, thereby filling a crucial gap in the design and analysis of LRS workflows.

## Supplementary Material

btag009_Supplementary_Data

## Data Availability

No new data were generated or analysed in support of this research.
