# Supplementary material for: esloco: simulation-based estimation of local coverage in long-read DNA sequencing
Source: Bioinformatics. 2026 Jan 9;42(2):btag009. doi: 10.1093/bioinformatics/btag009 (PMC12866673; doi:10.1093/bioinformatics/btag009)
Supplement: btag009_Supplementary_Data [file btag009_supplementary_data.zip › SupplementaryFigures_Weich_esloco.pdf]

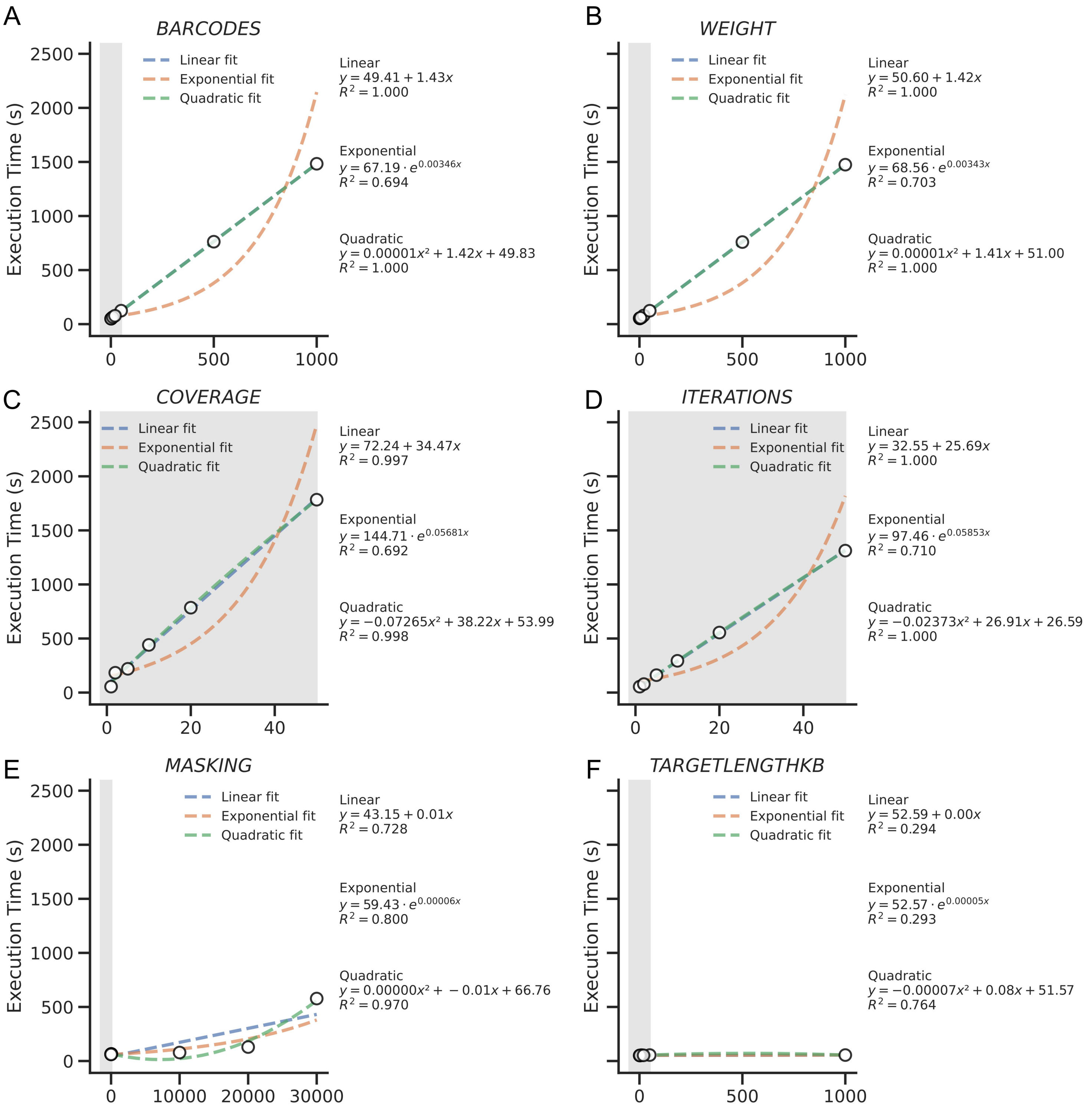

Figure S1. Speed benchmarking *esloco* and its parameters.

Grey boxes indicate the shared parameter range (n=1,2,5,10,20,50). Total runtime of *esloco* increases linearly with increasing numbers of (weighted) barcodes (**A**,**B**), coverage (**C**), and iterations (**D**). Runtime increases quadratically with increasing numbers of masked regions (**E**) with near-constant slopes until 20,000 regions. Runtime changes barely with increasing target space (in kb) (**F**).

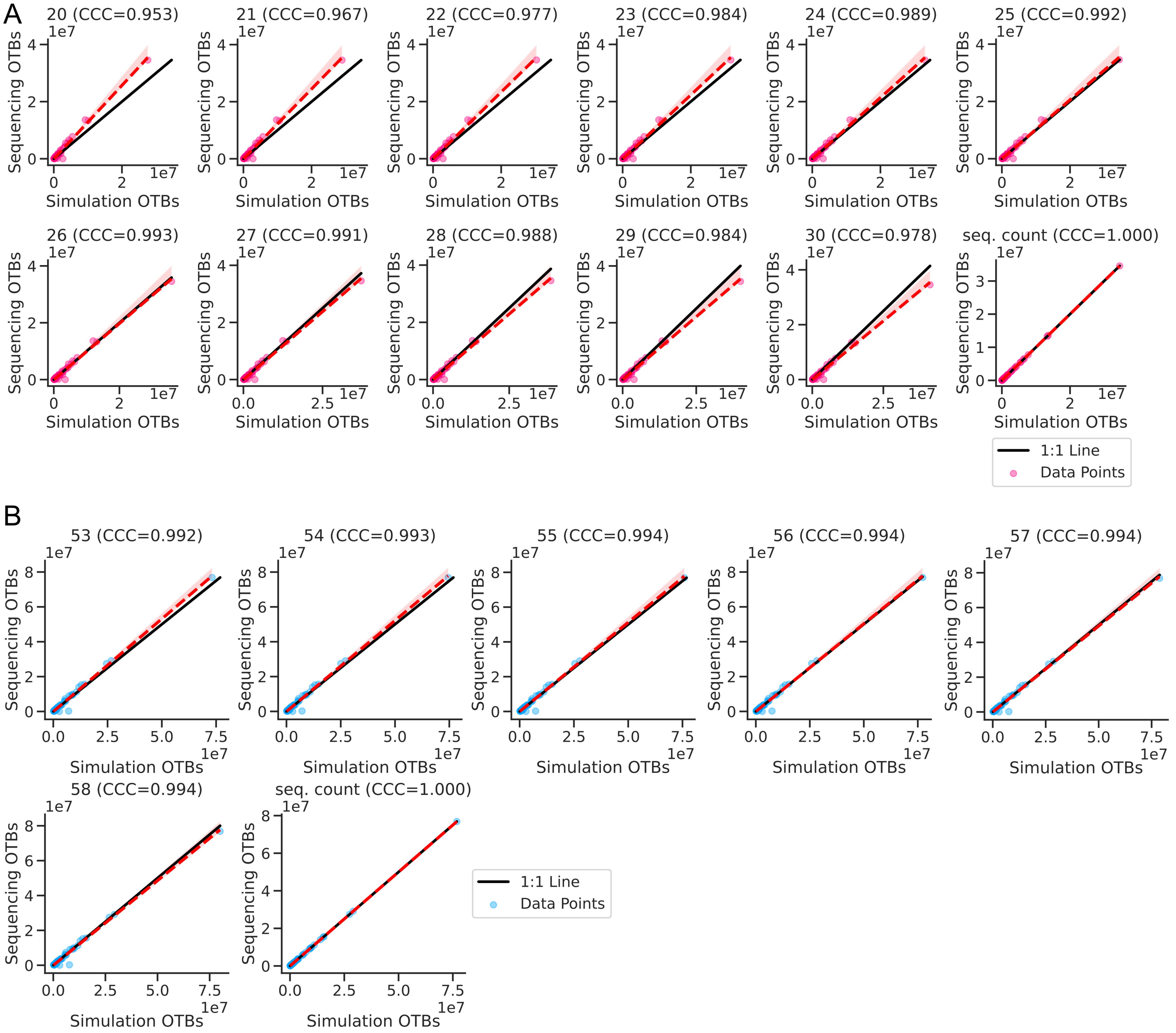

Figure S2. Concordance correlation coefficients (CCC) between simulated and empirical data for all simulated whole-genome coverage levels. A PacBio simulation benchmark. **B** Oxford Nanopore simulation benchmark.

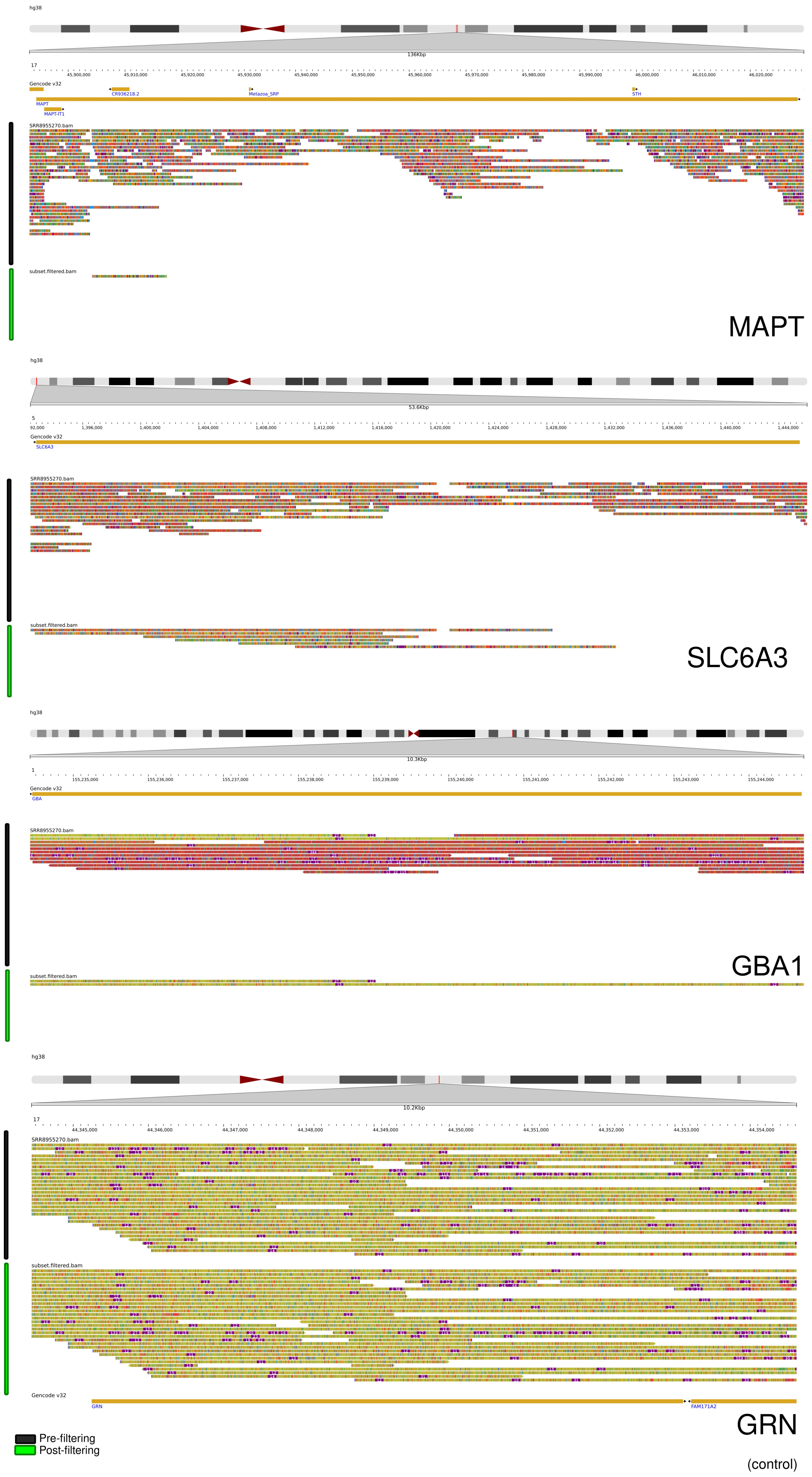

Figure S3. Read coverage of panel genes *MAPT*, *SLC6A3*, *GBA1*, and *GRN* in empirical data (PB) before (black) and after (green) filtering for mapping quality. Coverage of *MAPT*, *SLC6A3*, and *GBA1* was consistently overestimated in the simulation due to conservative mapping quality thresholds, whereas other panel genes, e.g, *GRN*, were less affected. Reads were visualized in IGV (v2.12.3).

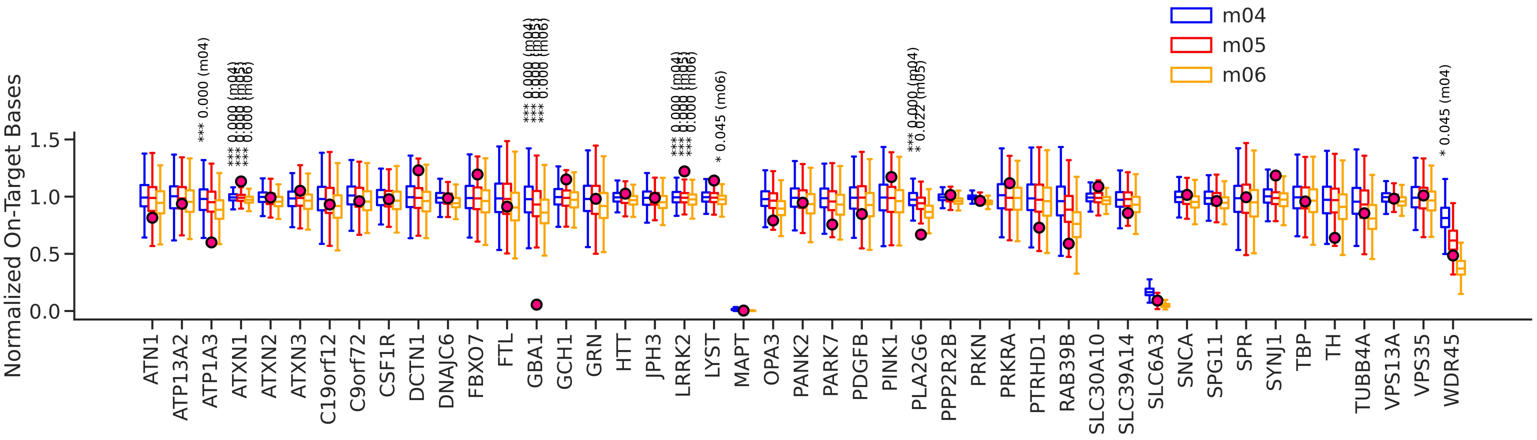

Figure S4. Predicted (boxplots) and observed (line) coverage in OTBs across panel genes using weighted masking. Across the panel genes, *esloco* estimated *MAPT*, *SLC6A3* and *WDR45* more accurately, although the third outlier, *GBA1*, remained consistently overestimated.Importantly, no new significantly different genes were introduced by the masking, underscoring *esloco*'s general robustness.

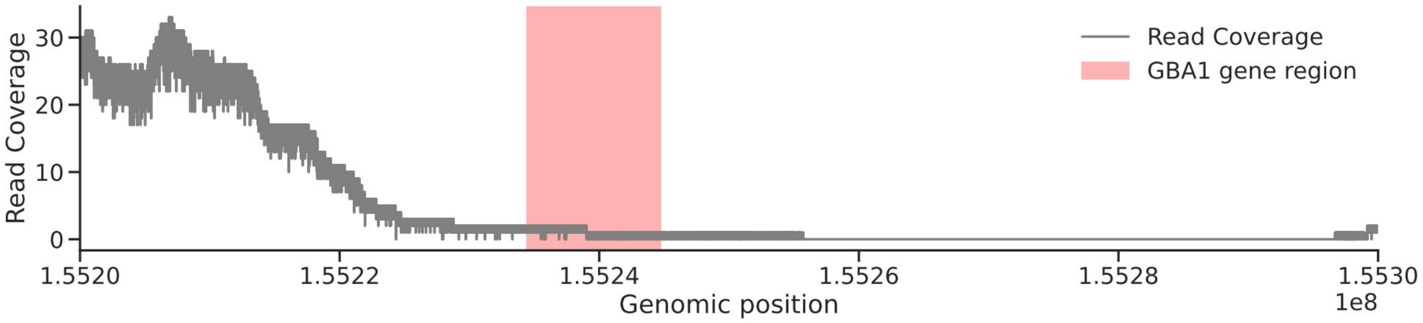

Figure S5. Read-coverage distribution for the 100 kb bin containing *GBA1*. The high variance in coverage indicates that the assigned blocking probability for this bin did not represent *GBA1* adequately, causing its persisting underestimation.
