# Supplementary material for: esloco: simulation-based estimation of local coverage in long-read DNA sequencing
Source: Bioinformatics. 2026 Jan 9;42(2):btag009. doi: 10.1093/bioinformatics/btag009 (PMC12866673; doi:10.1093/bioinformatics/btag009)
Supplement: btag009_Supplementary_Data [file btag009_supplementary_data.zip › ApplicationsNote_Weich_esloco_SupplementaryData.zip!/ApplicationsNote_SupplementaryData_Revision/Report/Case1_report.html]

Analysis Report


# Analysis Report

Results in ".../Case1\_report.html"...

### General Overview

### Match Overview

### Region-Specific Read Overlaps

### Coverage Overview

Previous
Next

### CPU and Memory Usage

### Simulation Configuration Parameters

```
[COMMON]
mode=ROI
reference_genome_path = hg38.fa
sequenced_data_path = SRR8955270.fasta.gz
output_path = Case1/
experiment_name = Case1
min_overlap_for_detection = 0
output_path_plots = Case1/
iterations = 1000
parallel_jobs = 50
no_cov_plots= False
coverages = [20,21,22,23,24,25,26,27,28,29,30]

[ROI]
roi_bedpath = parkinson_gene_panel.bed
```
